# Sulfamethoxazole Enhances Specific Enzymatic Activities under Aerobic Heterotrophic Conditions: A Metaproteomic Approach

**DOI:** 10.1021/acs.est.2c05001

**Published:** 2022-09-08

**Authors:** David M. Kennes-Veiga, Alba Trueba-Santiso, Valentina Gallardo-Garay, Sabela Balboa, Marta Carballa, Juan M. Lema

**Affiliations:** † CRETUS, Department of Chemical Engineering, University of Santiago de Compostela, Campus Vida, 15782 Santiago de Compostela, Galicia, Spain; ‡ CRETUS, Department of Microbiology, University of Santiago de Compostela, Campus Vida, 15782 Santiago de Compostela, Galicia, Spain

**Keywords:** activated sludge, antibiotics, biotransformation, metaproteomics, organic micropollutants, transformation products

## Abstract

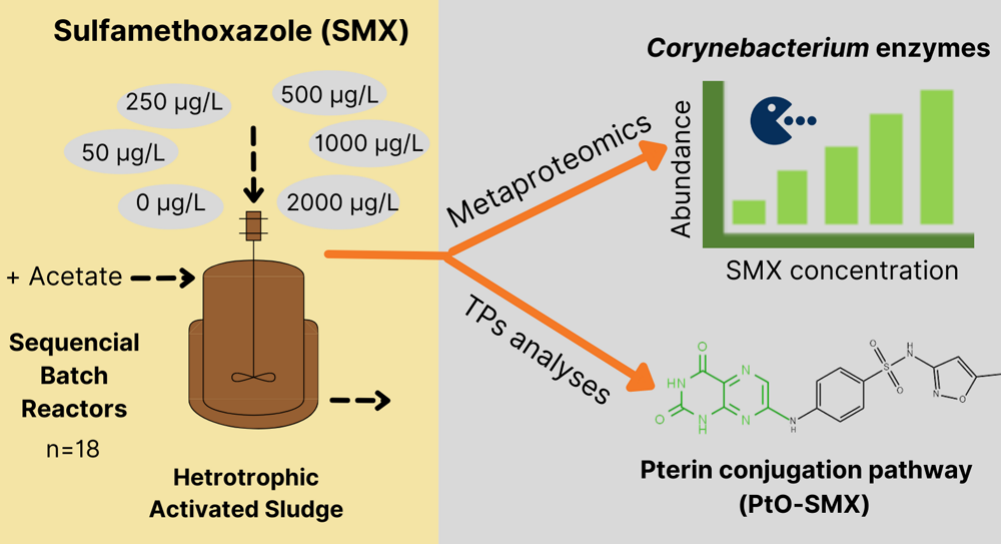

The growing concern about antibiotic-resistant microorganisms has focused on the sludge from wastewater treatment plants (WWTPs) as a potential hotspot for their development and spread. To this end, it seems relevant to analyze the changes on the microbiota as a consequence of the antibiotics that wastewater may contain. This study aims at determining whether the presence of sulfamethoxazole (SMX), even in relatively low concentrations, modifies the microbial activities and the enzymatic expression of an activated sludge under aerobic heterotrophic conditions. For that purpose, we applied a metaproteomic approach in combination with genomic and transformation product analyses. SMX was biotransformed, and the metabolite 2,4(1*H*,3*H*)-pteridinedione-SMX (PtO-SMX) from the pterin-conjugation pathway was detected at all concentrations tested. Metaproteomics showed that SMX at 50–2000 μg/L slightly affected the microbial community structure, which was confirmed by DNA metabarcoding. Interestingly, an enhanced activity of the genus *Corynebacterium* and specifically of five enzymes involved in its central carbon metabolism was found at increased SMX concentrations. Our results suggest a role of *Corynebacterium* genus on SMX risks mitigation in our bioreactors.

## Abstract 2

Metaproteomics allowed detection of an increase in the activity of the genus *Corynebacterium* with increased sulfamethoxazole concentrations in activated sludge under aerobic heterotrophic conditions.

## 1 Introduction

Antibiotics are a prominent group of organic micropollutants (OMPs) frequently detected in wastewater treatment plants (WWTPs) due to their increasing use in modern societies.^1,2^ As a consequence, WWTPs have become global hotspots for the development of antibiotic-resistant genes and bacteria, posing a serious environmental and health risk.^3−5^ Among antibiotics, sulfonamides are of particular importance because of their intensive utilization worldwide, with sulfamethoxazole (SMX) being the most broadly consumed one.^1,6^ In 2020, due to this growing concern, SMX was included in the “Surface Water Watchlist” published by the Water Framework Directive of the European Union to monitor and gather data about its potential risks to the aquatic environment.^7^

The typical SMX concentrations detected in the effluents of urban WWTPs range between 10 and 2000 ng L^–1^, with removal efficiencies varying between 30–75% depending on the influent concentration, the treatment processes applied, and the environmental conditions.^1,8−10^ Previous studies have highlighted the importance of heterotrophic microorganisms over nitrifiers to reduce SMX concentrations,^11^ and higher heterotrophic activities have shown to improve SMX biotransformation rates,^12^ suggesting that co-metabolism is the main responsible mechanism during SMX biotransformation in activated sludge systems. Additionally, the presence of acetate was shown to promote mineralization of SMX in a pure culture of *Achromobacter denitrificans* under aerobic heterotrophic conditions.^13^

Several biotransformation routes have been described for SMX, mostly using isolated microbial strains.^14−16^ For instance, certain *Microbacterium*, *Arthrobacter*, and *Leucobacter* strains (order *Actinobacteria*) have proven to metabolize SMX involving the *ipso*-hydroxylation sulfonamide biotransformation pathway.^17,18^ Also, SMX biotransformation through conjugation, oxidation, and hydrolysis reactions has been reported both with pure strains and in activated sludge systems.^6,19,20^ From these reactions, a wide range of transformation products (TPs) have been described and detected in WWTP effluents, many of them still possessing antibacterial activity and the capacity to be backtransformed to SMX.^6,21,10^ However, the knowledge on the microbial mechanisms involved in SMX biotransformation in activated sludge systems is still very limited.

In this sense, metaproteomics (or environmental proteomics) offers a suitable approach as it provides a global view of the proteins expressed by a microbial community at a specific moment,^22^ allowing us to study biological processes in their native environment while avoiding the time-consuming labor of isolating microorganisms.^23,24^ Although the analysis of highly complex samples, such as activated sludge, is quite challenging, recent advances in wastewater metaproteomics have partially overcome these limitations.^23,25−27^ Moreover, the potential of metaproteomics to study microbial community structures was also recently highlighted by Kleiner et al.^28^ and Wang et al.^24^ and applied to activated sludge by Azizan et al.^27^

The goal of this study was to obtain a better insight into the interaction of a selected range of SMX concentrations with a heterotrophic activated sludge microbial community. Specifically, we explored how increasing SMX concentrations affects the biotransformation capacity, the taxonomic composition of the community, and the bacterial enzymatic expressions. For that purpose, we applied a metaproteomic approach combined with genomic and transformation products analyses.

## 2 Materials and Methods

### 2.1 Sequential Batch Reactors

A total of 18 sequential batch reactors with a working volume of 100 mL were operated for 25 days in an incubator at neutral pH, 25 °C, 150 rpm, and sufficient oxygen concentrations (between 3–5 mg O_2_ L^–1^). Six different SMX concentrations were tested in triplicates: 0 (control), 50, 250, 500, 1000, and 2000 μg L^–1^. The reactors were inoculated with activated sludge from a WWTP near Santiago de Compostela (Spain), designed for 185,000 population equivalents, receiving an influent with a chemical oxygen demand ranging between 0.2 and 0.7 g L^–1^, and operated with hydraulic and sludge retention times of approximate 8 h and 10 d, respectively. The synthetic feeding consisted of a mixture of sodium acetate, acetic acid, ammonium chloride, potassium dihydrogen phosphate, sodium bicarbonate, calcium chloride, and magnesium sulfate (Table S1). The acetate concentration in our experiments was carefully designed in a previous test to ensure its presence during a major part of the bioreactor’s operation (data not shown). Additionally, other trace metals were added to promote microbial growth (Table S1), as well as allylthiourea (ATU) at a concentration of 5 mg L^–1^ to prevent nitrification. SMX was obtained from Sigma-Aldrich (Germany), and stock solutions were prepared in HPLC-grade methanol and stored at −20 °C.

The reactor’s operation was maintained under sterile conditions, and influent and effluent samples were taken over time to determine conventional parameters—total suspended solids (TSS); volatile suspended solids (VSS); concentrations of ammonium (NH_4_^+^), nitrate (NO_3_^–^), nitrite (NO_2_^–^) and oxygen; pH; and temperature—following standard methods.^29^ Additionally, acetate concentration was determined daily through gas chromatography using a DB-Wax-Agilent Technologies column (30 m × 0.250 mm × 0.25 μm), and samples from the feeding and the exhausted supernatant of the reactors were taken on days 2, 17, and 25 for SMX and TPs analysis.

### 2.2 Bioreactor’s Operation

On a daily basis, the content of the sequential batch reactors was centrifuged at 6000 rpm and 10 °C for 10 min to separate the biomass from the supernatant. Then, the exhausted supernatant was removed, and new feed was added together with the spike of SMX corresponding to each reactor. The objective of this operation was to minimize the accumulation of transformation products that could affect the biotransformation of raw SMX. Finally, the flasks were placed again in the incubator to resume operation. The entire process was performed under a fume hood to ensure sterile conditions.

### 2.3 SMX and TP Analyses

Samples were centrifuged at 6000 rpm and 10 °C for 10 min, and the obtained supernatant was prefiltered (AP4004705, Millipore) and filtered at 0.45 μm (HAWP04700, Millipore). Then, solid-phase extraction (SPE) was performed using 60 mg Oasis HLB cartridges (Waters Corp.) as described by Fernandez-Fontaina et al.,^30^ with volumes of 200 mL for the feeding samples and 100 mL for those of the reactors.

The quantification of SMX was performed using an Agilent G1312A liquid chromatograph with a binary pump and automatic injector HTC-PAL (CTC Analytics) connected to a mass spectrometer API 4000 triple quadrupole (Applied Biosystems). For TP detection, the samples were again analyzed by ultrahigh-performance liquid chromatography (UHPLC ELUTE, Bruker) coupled with quadrupole-time-of-flight mass spectrometry (Q-TOF-MS). Full-scan MS spectra were obtained in positive mode, and the acquisition of MS2 fragmentation spectra was triggered at *m/z* values corresponding to suspected TPs, which were selected based on previously determined TPs in the literature,^6,10,15,17,18,21,31−38^ the EAWAG pathway prediction system,^39^ and a list created manually by applying a range of plausible atomic modifications, such as hydroxylation, dealkylation, decarboxylation, deamination, conjugation, etc. The software TASQ (Bruker) was used to process the acquired data. SMX and TP analyses were done at the Santiago de Compostela University Mass Spectrometry and Proteomics facilities.

### 2.4 Metaproteomic Analyses

#### 2.4.1 Proteome Extractions

Proteome extractions were performed separately from 1 mL of homogenized samples collected from the inoculum and from each of the 18 sequential batch reactors on day 25. Cells were harvested by centrifugation at 6000 rpm, washed twice with PBS buffer, and subjected to 90 °C digestion for 20 min in 1% SDS Tris–HCl extraction buffer. Then, a physical lysis was performed by 12 min of beating with glass beads in a cell disruptor. Centrifugation at 3000 rpm was applied to remove cell debris and glass beads. Proteins were then precipitated with acetone in two consecutive steps at −20 °C and further resuspended with molecular-grade water. Protein concentration was measured using a bicinchoninic acid assay (BCA) assay kit (Thermo Fisher) at 540 nm upon calibration with a bovine serum albumin (BSA) standard curve (Table S5). Finally, triplicates were pooled together to obtain one mixed sample corresponding to the inoculum and each SMX treatment. The quality of the proteome samples was confirmed using SDS-PAGE electrophoresis with 4–12% Bis-Tris acrylamide NuPAGE gels (Thermo Fischer). A more detailed electrophoresis protocol can be found in Figure S1.

#### 2.4.2 Protein Identifications

Proteins were identified with a shotgun metaproteomic approach after “in-solution tryptic digestion” of the proteome samples.^25^ For this, samples were reduced, alkylated, trypsin-digested, and acidified. The digested samples were then desalted, vacuum-dried, and reconstituted in water with 2% acetonitrile (ACN) and 0.1% formic acid (FA). The obtained peptide mixtures (200 ng) were analyzed in a nanoElute (Bruker) nano-flow liquid chromatograph (LC) equipped with a C-18 reverse-phase column coupled to a high-resolution TIMS-QTOF (timsTOF Pro, Bruker) with a CaptiveSpray ion source (Bruker) at the Proteomics Platform-Proteored-ISCIII from the Biomedicine Research Institute of A Coruña (INIBIC). After ESI ionization, peptides were analyzed in data-dependent mode with parallel accumulation–serial fragmentation (PASEF) enabled. All the details regarding protein detection methodology are presented in the Supporting Information.

#### 2.4.3 Protein Data Analysis

Mass spectrometry raw files were processed with PEAKS Studio 10.6 build 20201221 (Bioinformatics Solutions Inc.). The MS/MS spectra were matched to in silico derived fragment mass values of tryptic peptides against the UniProtKB/Swiss-Prot database (release 2021_02). The detailed protocol for protein data analyses is presented in the Supporting Information. The mass spectrometry proteomics data was deposited in the ProteomeXchange Consortium via the PRIDE^40^ partner repository with the dataset identifier PXD029711 and 10.6019/PXD02971.

Inoculum samples were analyzed independently, while samples from day 25 were analyzed as a batch using the “Compare” semi-quantification module from PEAKS. The value Spec is based on peptide spectrum matches (PSM) and was used as indicator for the relative abundance of the proteins in each sample.^41^ The proteins identified with less than two unique peptides were excluded from all analyses, and for the taxonomic approach, the proteins were grouped at the genus level. The obtained list of peptide sequences was additionally processed with the UniPept Desktop v.1.2.1 for molecular function categorization. To explore changes in the expression of any enzyme that could be linked to SMX, the list of identified proteins was analyzed following two different strategies: (i) search for enzymes previously linked to the biotransformation of SMX by other authors (listed in Table S2) and (ii) search for enzymes differentially expressed at increased SMX concentrations.

### 2.5 DNA Metabarcoding

Genomic DNA from homogenized 1 mL samples of each bioreactor on day 25 was extracted using the Nucleospin Microbial DNA extraction kit (Machery-Nagel) according to the instructions of the manufacturer. Triplicates from each SMX concentration were pooled together after quantification and quality control with a Nanodrop and a Qubit fluorometer (Thermo Fisher). The V3–V4 hypervariable region for *Bacteria* was amplified using Bakt_341F (5′ CCT ACG GGN GGC WGC AG 3′) and Bakt_805R (5’ GAC TAC HVG GGT ATC TAA TCC 3′).^42^ DNA metabarcoding analyses of the region were carried out by AllGenetics and Biology SL (www.allgenetics.eu) in an Illumina PE150 platform.

Bioinformatic analysis was performed using the Microbial Genomics module (version 21.1) workflow of the CLC Genomics workbench (version 21.0.3). Raw sequences were filtered to remove low-quality reads and then clustered into Operational Taxonomic Units (OTUs) at 97% cutoff for sequence similarity and classified against the non-redundant version of the MiDAS 4 database.^43^ Only the most abundant bacterial OTUs (above 1% of the total observed OTUs) were considered.

## 3 Results

### 3.1 SMX Biotransformation and TP Identification

The presence of SMX in the sequential batch reactors, even at the highest concentration tested (2 mg/L), did not affect the consumption of the primary carbon source. Acetate removal was constant at approximately 140 mg L^–1^ h^–1^ (Table S3), while the average SMX biotransformation ranged between 62 and 78%, depending on the spiked concentration (Table 1) and showing clear characteristic trends. First, regardless of the SMX influent concentrations, the biotransformation yield increased from day 2 to day 17 and decreased on day 25 to values even lower than those observed on day 2. Second, on days 2 and 25, lower initial SMX concentrations lead to higher biotransformation yields, while this trend was not observed on day 17.

**Table 1:** SMX Biotransformation Yield in the Sequential Batch Reactors throughout the Experiment^a^

	Biotransformation (%)			
Influent SMX concentration (μg/L)	day 2	day 17	day 25	average
50	80	86	68	78 ± 8
250	77	87	63	76 ± 10
500	72	79	60	70 ± 8
1000	70	79	36	62 ± 19
2000	63	86	43	64 ± 18

a Yields were calculated based on 1 day removal data.

Nitrite and nitrate were never detected in the experiments, confirming that nitrification was efficiently inhibited by ATU (see Table S4). The SMX concentration retained on the activated sludge used in this study was analyzed and found to be negligible (data not shown), and therefore, we attribute the elimination of SMX in our experiments to biotransformation by heterotrophic biomass.

In this study, the TP 2,4(1*H*,3*H*)-pteridinedione-SMX (PtO-SMX) (Figure S5), which belongs to the pterin-conjugation pathway, was found in all reactors spiked with SMX at all sampling points except for the samples taken on day 2 from the reactor fed with 50 μg L^–1^ of SMX.

### 3.2 Inoculum Proteome Analyses

The high bacterial diversity in the sludge microbiome is reflected in the fact that the identified proteins were assigned to 122 different bacterial genera (Figure S2) and in that the genera that represented ≤1% abundance accounted for 30.57% of the total proteome (Table S5 and Figure S1). The genus with the highest protein contribution (17.83%) was *Burkholderia* (*c_Betaproteobacteria*, *o_Burkholderiales*). The next most abundant genera were *Cupriavidus*, *Bordetella*, *Paraburkholderia* (*c_Betaproteobacteria*, *o_Burkholderiales*), and *Rhodopseudomonas* (*c_Alphaproteobacteria*, *o_Rhizobiales*), all of them from the *Proteobacteria* phylum and with abundances ranging between 3.82 and 5.73%.

### 3.3 Effect of SMX Concentrations in the Metaproteome

A total of 1051 proteins from 114 bacterial genera were identified in the analysis of the proteome samples collected on day 25 of the bioreactor’s operation (Table S6). The proteins identified were mostly related to cell maintenance, translation, ATPase activity, and the tricarboxylic acid cycle (TCA) (Figure S4). None of the enzymes previously linked in the literature to SMX biotransformation were detected in this study.

**Figure 1:**
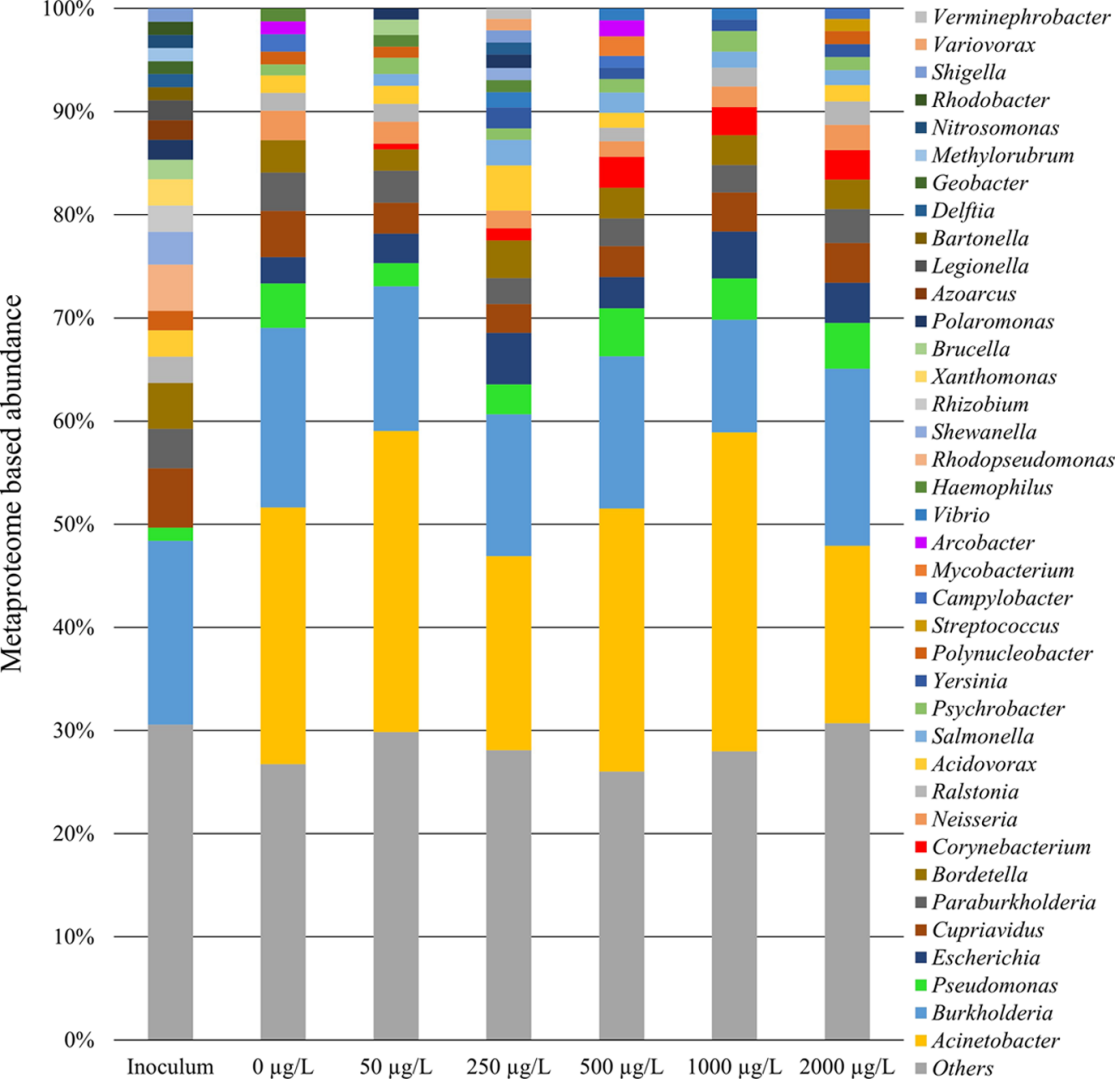
Abundance of the bacterial genera identified in the metaproteomic analyses of the inoculum and biomass samples collected on day 25 from the bioreactors fed with different SMX concentrations. The genera identified with a contribution ≤1% are grouped in Others.

Figure 1 presents the contribution of each bacterial genus in the different SMX treatments in comparison with the inoculum. Genera with a contribution of ≤1% of the total were grouped in Others and accounted for 25–32% of the total.

**Figure 2:**
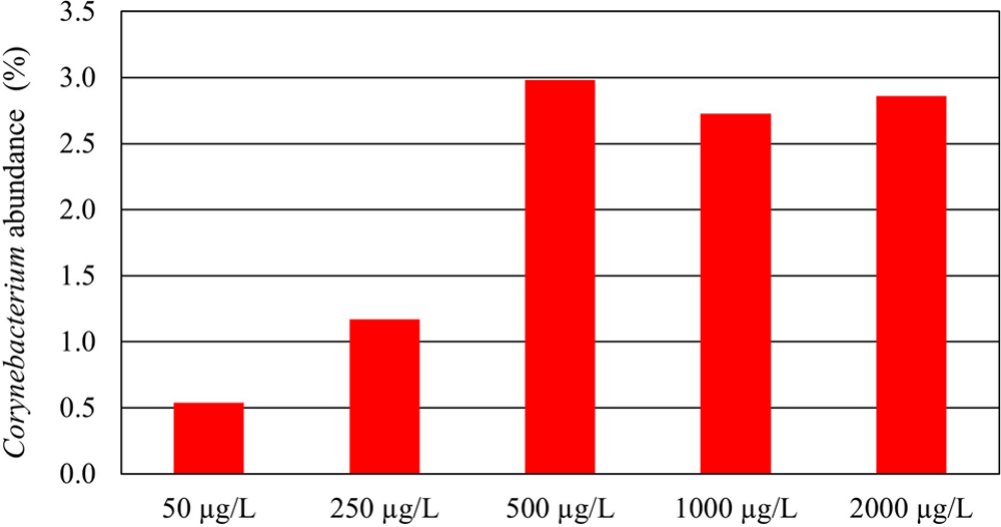
*Corynebacterium* abundance determined through shotgun metaproteomics in the microbial community on the bioreactors spiked with different SMX concentrations at day 25. At the inoculum and 0 μg L^–1^ SMX, *Corynebacterium* proteins were detected in <1% abundance.

The protein contribution of *Burkholderia* remained constant in all reactors and similar to the inoculum. Differently, a marked increase in *Acinetobacter* was detected in all reactors compared to the inoculum, including those without SMX addition, which was attributed to the operational conditions (e.g., presence of ATU) and acetate consumption. The proteins from these two genera dominated the proteomes, accounting jointly for 32.55–43.44%. Interestingly, there was an increase in the abundance of the genus *Corynebacterium* linked to the SMX concentration up to 500 μg L^–1^, and then it remained constant (Figure 2). Moreover, seven enzymes from the genus *Corynebacterium* were differentially expressed in the presence of varying SMX concentrations (Table 2): isocitrate lyase, aconitate hydratase, malate dehydrogenase, citrate synthase (related with TCA cycle) enolase (glycolysis), elongation factor EfTU, and 50S ribosomal protein L22.

**Table 2:** Total Number of Peptides and Unique Peptides, and Spec Value of the Enzymes Assigned to the Genus *Corynebacterium* and Differentially Expressed at Different SMX Concentrations, Detected by Shotgun Metaproteomics at Day 25

Protein ID	Total peptides detected	Total unique peptides detected^a^	0 μg/L^b^	50 μg/L	250 μg/L	500 μg/L	1000 μg/L	2000 μg/L
elongation factor (Ef_TU)	22	6	4	18	28	29	29	32
50 S ribosomal protein L22	12	12	7	19	18	85	40	59
isocitrate lyase	10	10	0	8	13	18	15	24
aconitate hydratase	7	6	0	0	1	12	22	4
malate dehydrogenase	6	6	0	0	4	8	18	9
enolase	5	5	0	0	4	13	9	3
citrate synthase	5	4	0	0	2	9	4	3

a Unique peptides are considered unique to a protein group.

b Spec values are shown for each SMX concentration. Spec values are based on the spectral peptide match counts and are presented here for comparison of the relative abundance of proteins in the samples.

### 3.4 Community Structure Based on DNA Metabarcoding

**Figure 3:**
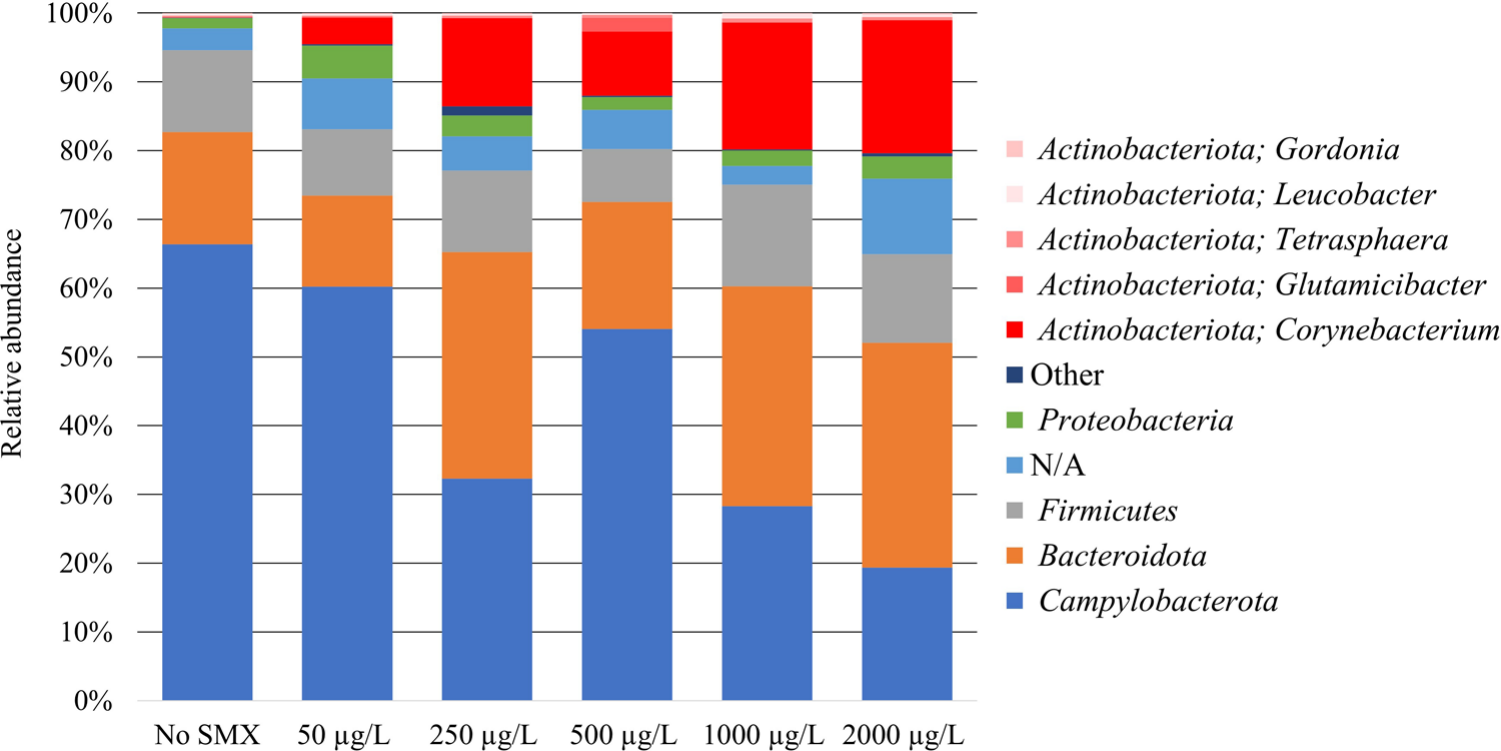
Taxonomic composition of the microbial communities on day 25 of operation of the reactors fed with different SMX concentrations according to DNA metabarcoding. Results are shown at phylum level except for genera belonging to *Actinobacteriota*, which are displayed individually. Phyla representing ≤1% abundance are clustered as Other, while N/A refers to the population that was not taxonomically assigned.

The results obtained by DNA metabarcoding (Figure 3) showed an increase in the abundance of *Actinobacteriota* phylum related in a positive manner to SMX concentration. Genera from this phylum are displayed individually in Figure 3. Among them, *Corynebacterium* was the predominant under all SMX concentrations, and its relative abundance increased in highest SMX concentrations.

Conversely, the addition of SMX negatively affected *Campylobacterota*, which reduced its relative abundance from 67% in the absence of SMX to 20% in the 2000 μg SMX L^–1^ treatment. *Proteobacteria* and *Firmicutes* phyla were not significantly affected by SMX, while *Bacteroidota* increased their abundance with SMX.

## 4 Discussion

The results obtained in this study confirmed the high capability of the heterotrophic sludge to biotransform SMX (Table 1), as previously shown in the literature.^31,11,44^ Moreover, the presence of SMX under the concentrations tested (50–2000 μg L^–1^) did not negatively affect acetate consumption. This might have two possible explanations: (i) the highest concentration tested (2000 μg L^–1^) is still below inhibitory levels, and (ii) the role of specific bacteria in SMX biotransformation mitigated the potential negative effects of the antibiotic over other microorganisms. Additionally, the biotransformation yield followed a noteworthy tendency both with time and increased SMX concentrations. The improved biotransformation on day 17 compared to day 2 as well as the reduced biotransformation yield of SMX on day 2 at higher initial concentrations can be related to the acclimation phase of the microorganisms capable of biotransforming SMX. Their higher abundance on day 17 might have allowed them to reach a constant biotransformation extent in the 80–90% range in all bioreactors. This agrees with the findings of Li et al.,^45^ who, after an extended lag phase proportional to the doses amended, observed the biotransformation of multiple antibiotics by bacteria from different genera. The decreased biotransformation on day 25, intensified at higher SMX concentrations, is in line with a recent study from Achermann et al.^46^ The authors found a negative correlation between higher solid retention times and sulfonamide biotransformation among the 42 micropollutants tested at their bioreactors. These observations might be associated to the accumulation of TPs. The SMX sorption to activated sludge is negligible regarding our experiments, in line with previous works^13^ and supported by the physical–chemical characteristics of this compound: low log solid–water distribution coefficient (log *K*_d_ = 0.8–1.8 in digested sludge at different operational conditions) and low *n*-octanol–water distribution coefficient (*K*_now_ = 0.89).^47^ However, some TPs from SMX biotransformation pathways previously reported on the literature might present different characteristics that leads to sorption to the biomass (e.g., 3-amino-5-methylisoxazole (3A5MI)^37^). This accumulation could limit the biotransformation of the parent compound due to reversibility reaction events,^48^ thermodynamic limitations,^49^ or by exerting toxicity over a certain concentration,^50,51^ thus outweighing the increased presence of SMX degraders.

Both metaproteomic (Figure 2) and genomic (Figure 3) results show an increase in *Corynebacterium* activity positively related with SMX concentration, revealing that members of this genus might possess an advantage over the other members of the community. The protein contribution of this genus to the total of proteins identified and, specifically, the abundance of five enzymes related to their central carbon metabolism increases up proportionally to 500 μg L^–1^ SMX treatment, decreasing then at 1000 and 2000 μg L^–1^ (Table 2). These results correlate with the SMX biotransformation yield trend observed in the reactors, suggesting that *Corynebacterium* strains present in our experiments play a role in SMX biotransformation. Such a link has been observed for closely related members of its phylum, *Actinobacteria*,^52^ as for instance, the genera *Microbacterium*,^18^*Arthrobacter*, *Achromobacter*, *Leucobacter*,^52^ or *Gordonia*,^35^ which belongs to the same order as *Corynebacterium* (*Mycobacteriales*).

Among the different TPs reported for SMX biotransformation in activated sludge systems (Table S2), only PtO-SMX (Figure S5) was found in the sequential batch reactors fed with SMX. This TP has been previously described both in lab-scale studies with activated sludge and in WWTPs effluents.^6^ The fact that it was not detected in the reactor fed with 50 μg L^–1^ of SMX on day 2 is attributed to its expected low concentration in this sample. The detection of a pterin conjugate indicates that this SMX biotransformation route was active on the microbiome. The formation of PtO-SMX happens when sulfonamides interact with the enzyme dihydropteroate synthetase (DHPS), hindering folic acid synthesis through competitive inhibition. The pathway consists of SMX conjugation and oxidation to pterin-SMX and its subsequent hydrolysis to PtO-SMX, which is further transformed following various unclear steps possibly involving oxidation and decarboxylation reactions.^6^ However, the enzymes involved in the SMX pterin-conjugation pathway and, particularly, pterin deaminase, which catalyzes the biotransformation of pterin-SMX to PtO-SMX, were not detected in the present study. This can be caused by their expected low relative abundance in comparison to housekeeping-related proteins or those involved in central carbon metabolism. Different than other molecular techniques, such as transcriptomics, metaproteomics are biased to the most abundantly expressed proteins in the mixed sample as they lack the amplification step of the polymerase chain reaction (PCR).

Most of the overexpressed *Corynebacterium* enzymes are related to the TCA cycle. As *Corynebacterium* seems to be more resistant to SMX than other members of the microbial community, its contribution to acetate transformation should be higher at the increased SMX concentrations. Moreover, previous data in the literature suggest a potential role of TCA cycle enzymes in the metabolism of sulfonamides.^13,17,53,54^ Both facts could explain the overexpression of TCA enzymes. Their typical substrate-specificity makes unlikely their involvement in the initial steps of SMX biotransformation. However, as per the previous literature, they could participate in the conversion of smaller metabolites from further steps. Interestingly, the sulfonamide TP 4-aminophenol was previously shown to be channeled into the TCA cycle via 1,2,4-trihydroxybenzene or hydroquinone in *Microbacterium* sp. strain *BR1*,^18,53,54^ and the same underlying mechanism was suggested for other bacteria obtained from WWTPs and capable of mineralizing SMX.^35,54^ Related with this, a link between the TCA cycle and SMX mineralization was previously reported in Nguyen et al (2017).^13^ The authors found acetate as a biogenic substrate that improved SMX degradation kinetics at concentrations ranging from 600 ng/L to 150 mg/L in a pure culture of *Achromobacter denitrificans* PR1 capable of using SMX as sole source of carbon, nitrogen, and energy at higher concentrations.

Taking all results together, the most feasible interpretation is that the *Corynebacterium* spp. present in our bioreactors possess an advantage over the other members of the bacterial community, which might rely on their highest metabolic activity (TCA cycle) being able to produce more dihydropteroate synthase. This would be in line with the mechanism of survival to sulfonamides termed transformation in Nunes et al.^17^ On it, the parent compound is not degraded but transformed, and although this process may be associated with bacterial growth, it occurs in co-metabolism (i.e., in the presence of additional carbon and energy sources). The definition of co-metabolism applied here is that the highest consumption of a main substrate leads to the transformation of a secondary one. The isolation of the *Corynebacterium* spp. present in the reactors would be of great interest to individually evaluate its biotransformation capacities. Nonetheless, it might also be considered that the behavior of the microorganisms in pure culture might differ from what happens in real environments.

## 5 Implications

This study highlights the capacity of the heterotrophic activated sludge to biotransform SMX, suggesting that even the novel, more energy-efficient WWTPs operating at high organic loads and short sludge retention times should be capable of reducing SMX influent concentrations.

The main heterotrophic activity (acetate consumption) in the reactors was not affected under the tested SMX concentrations, while the composition of the microbiota slightly did. Our results pointed toward the key role of *Corynebacterium* to maintain the fitness of this microbial community. SMX survival in *Corynebacterium* seemed linked to the TCA cycle, highlighting the need to dedicate research efforts to elucidate the involvement of central metabolic proteins in the removal mechanisms of OMPs.

Dihydropteroate synthase and pterin deaminase were not detected, indicating that further development of the applied techniques to lower the identification thresholds or to reduce sample complexity is still required. Nevertheless, thanks to the combination of metaomics and transformation product analyses, this work provides new insights on the effect of SMX on activated sludge under aerobic heterotrophic conditions. This fact confirms the advantages of including metaproteomic analysis to obtain a more realistic picture of a specific microbial environment and to find causal links between OMP biotransformation and the microbiological data.

## Supporting Information Available

Complete list of identified proteins in the inoculum and in the proteomes from the bioreactors at day 25 (ZIP)
Detailed protocol for protein detection; protein concentrations, SDS-PAGE gel images, bacterial genera, protein functions, and gene ontology (GO) molecular function categories from proteome samples; literature review of SMX transforming bacteria; feeding composition, average acetate consumption rates, and ion concentrations in the bioreactors and mass spectra for TPs screening (PDF)
