# Supplementary material for: Sulfamethoxazole Enhances Specific Enzymatic Activities under Aerobic Heterotrophic Conditions: A Metaproteomic Approach
Source: Environ Sci Technol. 2022 Sep 8;56(18):13152–9. doi: 10.1021/acs.est.2c05001 (PMC9686132; doi:10.1021/acs.est.2c05001)
Supplement: Supplementary file 2 — es2c05001_si_002.pdf [file es2c05001_si_002.pdf]

Supplementary information

Sulfamethoxazole enhances specific enzymatic activities under aerobic heterotrophic conditions: A metaproteomic approach

David M. Kennes-Veiga[1,‡], Alba Trueba-Santiso[1,‡,*], Valentina Gallardo-Garay[1], Sabela Balboa[2], Marta Carballa[1] and Juan M. Lema[1]

[1] CRETUS, Department of Chemical Engineering, University of Santiago de Compostela, Campus Vida, 15782, Santiago de Compostela, Galicia, Spain.

[2] CRETUS, Department of Microbiology, University of Santiago de Compostela, Campus Vida, 15782, Santiago de Compostela, Galicia, Spain.

[‡]The first two authors contributed equally to this work.

[*] Corresponding author. E-mail address: albamaria.trueba@usc.es. Tel.: +34 881816020.

Supplementary data contains:
18 pages
2 texts
5 tables
9 figures
References

Text S1. Detailed protocol for protein detection

Equal amounts of each sample pool (approximately 10 µg) were first reduced with 10 mM dithiothreitol for 1 h at 37 °C and subsequently alkylated with 50 mM iodoacetamide for 45 minutes at room temperature in the dark. The samples were digested with sequencing grade modified trypsin (Promega) at a 1:40 enzyme-to-substrate ratio. After 16 h of digestion at 37 °C, the samples were acidified with 10% trifluoroacetic acid until reaching approximately a pH 3. The digested peptides were desalted using in-house made stage tips (3M Empore SPE-C18 disk, 47 mm, Sigma Aldrich) and finally dried under speed-vacuum (Thermo Fisher). The dried eluates were reconstituted in water with 2% acetonitrile (ACN) and 0.1% FA for direct LC-MS. The peptide mixture (200 ng) was loaded in a nanoElute (Bruker) nano-flow LC coupled to a high-resolution TIMS-QTOF (timsTOF Pro, Bruker) with a CaptiveSpray ion source (Bruker). Liquid chromatography was performed at 50 °C and with a constant flow of 500 nL/min on a reversed-phase column (15 cm x 75 m i.d.) with a pulled emitter tip, packed with 1.9 m C18-coated porous silica beads (Dr. Maisch, Ammerbuch-Entringen). Chromatographic separation was carried out using a linear gradient of 5-35% buffer B (100% ACN and 0.1% FA) over 60 min. After ESI ionization, peptides were analyzed in data-dependent mode with the Parallel Accumulation–Serial Fragmentation (PASEF) enabled.

Text S2. Detailed protocol for protein data analyses

Mass spectrometry raw files were processed with PEAKS Studio 10.6 build 20201221 (Bioinformatics Solutions Inc.). The MS/MS spectra were matched to in silico derived fragment mass values of tryptic peptides against the UniProtKB/Swiss-Prot database (release 2021_02). The search parameters were: Parent Mass Error Tolerance: 15.0 ppm;

Fragment Mass Error Tolerance: 0.05 Da; Enzyme: Trypsin; Fixed Modifications: Carbamidomethylation; Variable Modifications: Acetylation (Protein N-term), Deamidation (NQ), Oxidation (M) and Acetylation (N-term); Maximum Variable Post Translational Modifications Per Peptide: 3. Matches were filtered for 1% FDR at the peptide level.

Table S1. Feeding composition used in the sequential batch reactors.

**Compounds**	**mg L^{-1}**
Sodium acetate ($CH_3COONa·3H_2O$)	4700
Acetic acid (CH_3COOH)	2170
Ammonium chloride (NH_4Cl)	1050
Sodium bicarbonate ($NaHCO_3$)	4800
Potassium dihydrogen phosphate (KH_2PO_4)	170
Calcium chloride ($CaCl_2$)	70
Magnesium sulfate ($MgSO_4$)	70
Iron (III) chloride $FeCl_3·6H_2O$	0.15
Boric acid (H_3BO_3)	0.015
Copper (II) sulfate ($CuSO_4·5H_2O$)	0.003
Potassium iodide (KI)	0.003
Zinc sulfate ($ZnSO_4·7H_2O$)	0.012
Cobalt chloride ($CoCl_2·6H_2O$)	0.015
Manganese (II) chloride ($MnCl_2·4H_2O$)	0.012

Table S2. Collection of bacteria described in literature with the capacity to biotransform sulfamethoxazole under aerobic conditions. In the cases where the enzymes/gene responsible for the biotransformation or the TPs were not determined, the symbol (-) was used.

**Bacterial Taxon**	**SMX Biotransformation Mechanism**	**Additional carbon source**	**SMX initial concentration (mg/L)**	**Enzymes/ genes involved in SMX biotransformation**	**Transformation Products**
Acinetobacter sp. W1[1]	Metabolism	None	5-240	EC 2.3.1.5: arylamine N-acetyltransferase;<br>EC 3.5.1.85: N-acetyl-1-phenylethylamine hydrolase;<br>monooxygenases or dioxygenases and amidases	4-N-(hydroxyl-methyl)-N-(3- amino-5-carboxyl) benzenesulfonamide<br>4-N-(hydroxyl-methyl- carboxyl)-N-(3-amino-5-carboxyl) benzenesulfonamide<br>4-hydroxy-benzenesulfonic acid<br>3-hydroxylamine-amino-5-carboxyl
Gordonia sp.[2]	Metabolism	None	5	EC 1.13.11.66: hydroquinone 1,2 dioxygenase	4-aminophenol<br>hydroquinone
Microbacterium sp. BR1[3,2]	Metabolism	None	250	sulfonamide monooxygenase (*sadA*);<br>4-aminophenol monooxygenase (*sad*B);<br>FMN reductase (*sad*C);<br>FMNH2-dependient monooxygenases; NADH-dependent monooxygenases	3-amino-5-methylisoxazole<br>4- aminophenol<br>1,2,4-trihydroxybenzene<br>hydroquinone
Labrys sp.[2]	Metabolism	None	5	EC 1.13.11.66: hydroquinone 1,2 dioxygenase	4-aminophenol<br>hydroquinone
Ochrobactrum sp.[2]	Metabolism	None	5	-	4-aminophenol<br>hydroquinone

Pseudomonas psychrophila HA-4[4]	Metabolism	None	100	-	aniline<br>3-amino-5-methylisoxazole<br>4- aminothiophenol<br>sulfanilamide
Achromobacter denitrificans PR1 *Leucobacter sp.* GP [5,6]	Metabolism/<br>Cometabolism	Without succinate/With succinate	0.5-150	sulfonamide monooxygenase (*sadA*);<br>4-aminophenol monooxygenase (*sad*B);<br>FMN reductase (*sad*C)	3-amino-5-methylisoxazole
Rhodococcus equi [7]	Metabolism/<br>Cometabolism	Without glucose/With glucose	6	EC 2.3.1.5: arylamine N-acetyltransferase;<br>EC 3.5.1.75: uretanase*;<br>EC 3.5.1.85: N-acetyl-feniletilamine hydrolase*	N4-acetyl- sulfamethoxazole
Activated sludge [8]	Cometabolism	None	0.25-0.4	EC 3.5.4.11: pterin deaminase	2,4(1H,3H)-pteridinedione-sulfamethoxazole
Ammonia oxidizing bacteria [9]	Cometabolism	Acetate	0..1	EC 1.14.99.39: ammonia monooxygenase	4-Nitro sulfamethoxazole<br>Desamino-sulfamethoxazole<br>N4-acetyl- sulfamethoxazole
Pseudomonas aeruginosa [7]	Cometabolism	Glucose	6	EC 2.3.1.5: arylamine N-acetyltransferase	-
Rhodococcus rhodochrous [10]	Cometabolism	Glucose	31.6	-	Hydroxy-N-(5-methyl-3-isoxazole) benzene-1-sulfonamide

*Enzymes that hydrolyze acetylated SMX by-products.

Table S3. Average acetate consumption rate in the sequential batch reactors fed with different SMX concentrations.

Initial SMX concentration (µg/L)	Acetate consumption rate (mg/L·h)
0	137
50	140
250	142
500	144
1000	139
2000	139

Table S4. Average concentrations of ions determined by ionic chromatography along the bioreactor's operation. Samples were taken from the supernatant at the end of each daily period, after centrifugation of the biomass.

Ions	Synthetic feeding	50 µg SMX/L	500 µg SMX/L	2000 µg SMX/L
SO_4^{-2} (mg/L)	815 ± 0.6	756 ± 1.8	754± 2.4	767 ± 3.5
NO_2^- (mg/L)	0 ± 0.0	0 ± 0.0	0 ± 0.0	0 ± 0.0
NO_3^- (mg/L)	0 ± 0.0	0 ± 0.0	0 ± 0.0	0 ± 0.0
NH_4^+ (mg/L)	549 ± 1.2	204 ± 2.7	150 ± 5.2	160 ± 8.3

Table S5. Protein concentration determined by BCA assay in proteome samples obtained from the inoculum and biomass samples from day 25 of the operation of the bioreactor.

Proteome sample	Protein concentration (µg/µL)	
	Average	Standard deviation
Inoculum	0.39	0.03
0 µg/L	1.74	0.01
50 µg/L	1.54	0.01
250 µg/L	1.85	0.02
500 µg/L	2.37	0.00
1000 µg/L	1.71	0.01
2000 µg/L	2.03	0.10

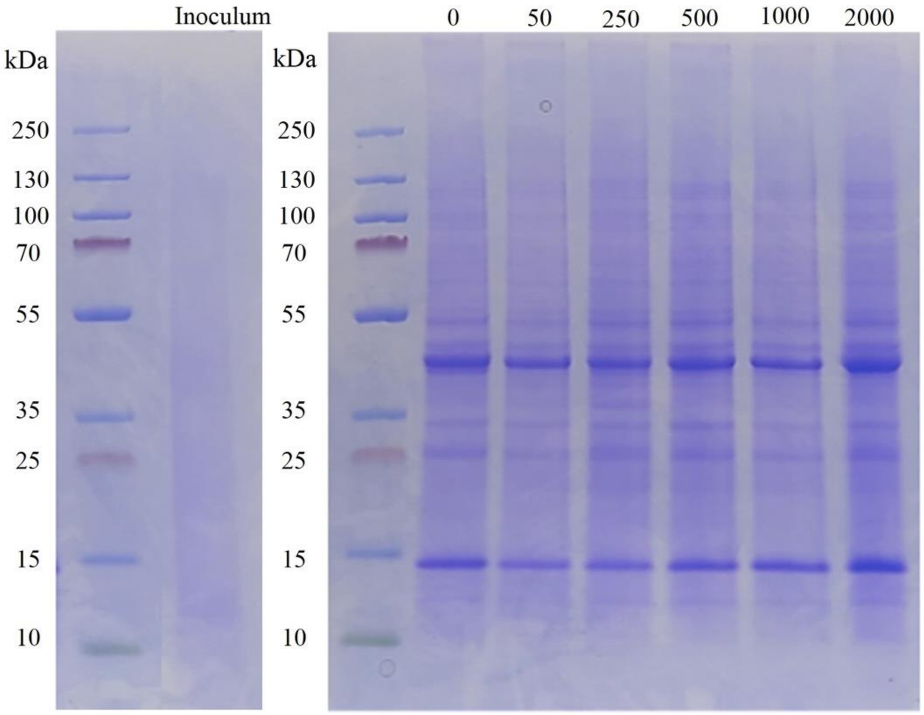

Fig. S1: SDS-PAGE gel electrophoresis of proteome samples from the inoculum and biomass from day 25 of the bioreactors operated with different SMX concentrations (0 – 2000 µg/L). All lanes were loaded with 15 µg protein aliquots, and as molecular weight ruler, 3 µL of 10-250 kDa prestained PageRuler Plus (ThermoFisher) were used. Electrophoresis was performed at 200 V for 30 minutes, with 4-12% Bis-Tris acrylamide NuPAGE gels and MES-SDS running buffer. Gels were prefixed and stained using a standard Coomassie R-250 staining protocol. Poor resolution of protein bands in the inoculum sample is attributed to the presence of humic or nucleic acids, or protein degradation in the sample [11].

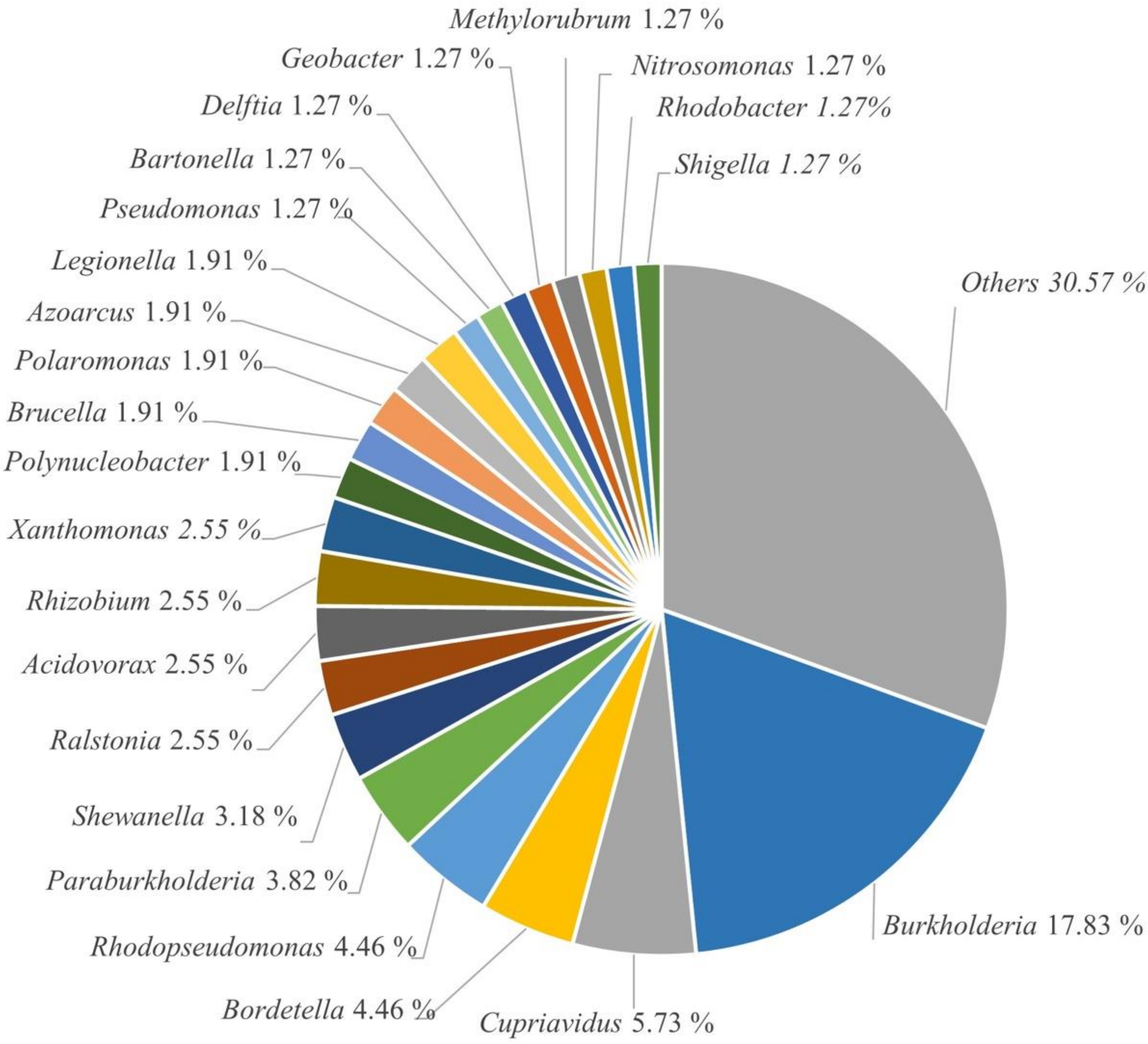

Fig. S2. Contribution of each bacterial genera to the total of proteins identified in the activated sludge sample used as inoculum in this study. Genera contributing with ≤1% to the total are grouped in Others.

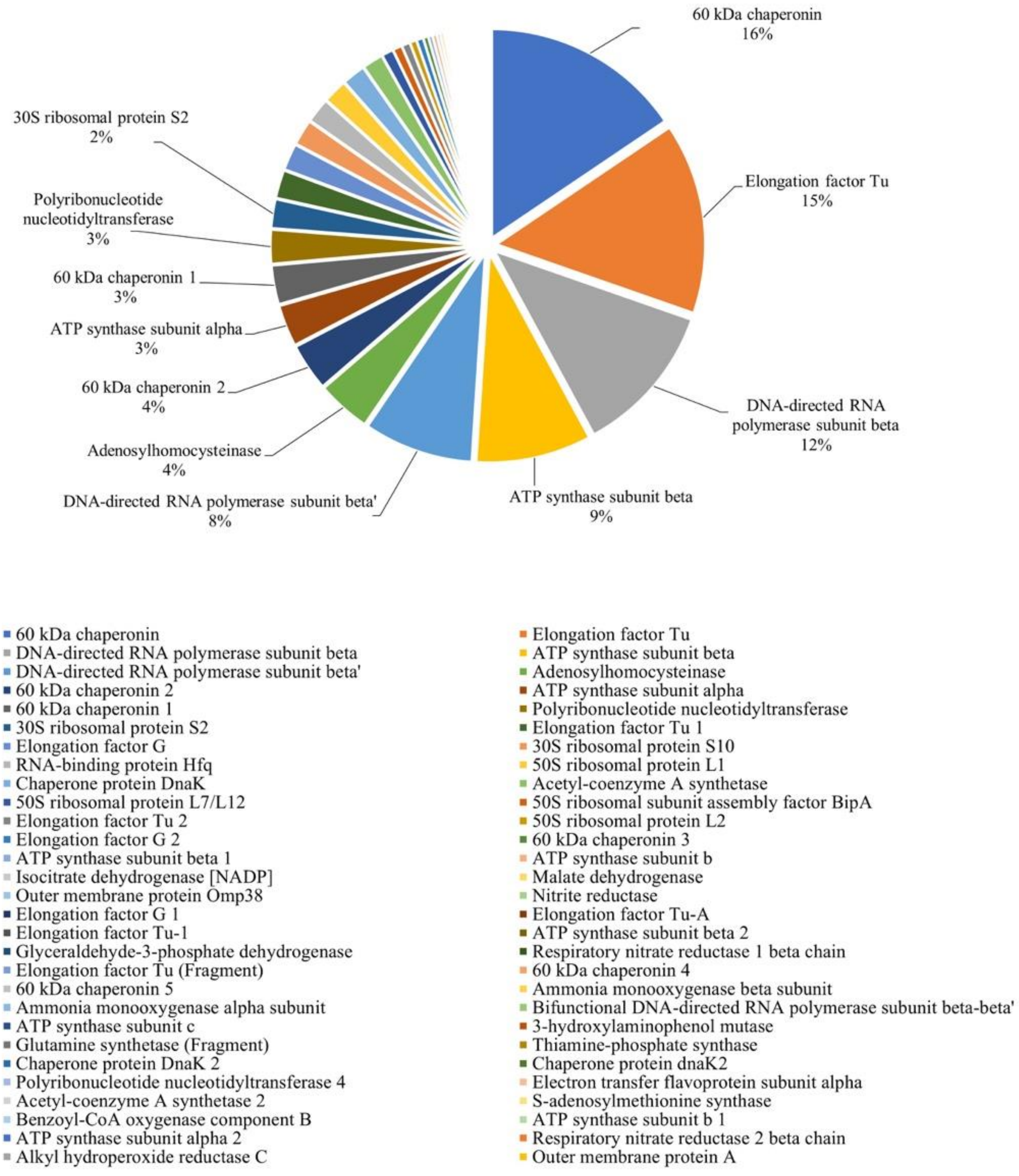

Fig. S3. Distribution of the protein functions of the proteins identified in the activated sludge sample used as inoculum in this study.

Name	GO-term	0 µg/L	50 µg/L	250 µg/L	500 µg/L	1000 µg/L	2000 µg/L
ATP binding	GO:0005524	354	408	449	490	390	350
structural constituent of ribosome	GO:0003735	269	305	333	357	312	231
rRNA binding	GO:0019843	203	221	239	253	229	164
metal ion binding	GO:0046872	150	174	208	235	181	155
GTP binding	GO:0005525	132	177	169	192	148	121
magnesium ion binding	GO:0000287	121	126	158	184	130	111
GTPase activity	GO:0003924	117	154	147	168	131	109
DNA binding	GO:0003677	107	126	135	179	115	89
translation elongation factor activity	GO:0003746	104	132	137	149	117	98
ATPase	GO:0016887	102	106	124	136	115	105
proton-transporting ATP synthase activity	GO:0046933	101	117	124	114	110	92
hydrolase activity	GO:0016787	98	116	121	115	107	90
proton-transporting ATPase activity	GO:0046961	85	98	109	94	95	82
unfolded protein binding	GO:0051082	83	89	92	100	94	81
DNA-directed 5'-3' RNA polymerase activity	GO:0003899	75	84	90	127	79	56
tRNA binding	GO:0000049	67	87	74	93	70	62
zinc ion binding	GO:0008270	59	75	68	106	64	51
RNA binding	GO:0003723	47	58	58	71	55	44
4 iron, 4 sulfur cluster binding	GO:0051539	39	41	54	57	46	38
NADP binding	GO:0050661	35	39	40	51	34	41
ribonucleoside binding	GO:0032549	31	30	33	43	28	21
NAD binding	GO:0051287	28	30	44	41	35	30
isomerase activity	GO:0016853	27	30	32	36	19	28
L-malate dehydrogenase activity	GO:0030060	23	25	31	33	36	26
aconitate hydratase activity	GO:0003994	22	26	27	35	31	27
citrate dehydratase activity	GO:0047780	22	26	27	35	31	27
ketol-acid reductoisomerase activity	GO:0004455	22	25	22	30	16	24
pyridoxal phosphate binding	GO:0030170	21	23	23	27	17	19
mRNA binding	GO:0003729	19	18	19	24	22	13
transferase activity	GO:0016740	18	21	19	27	21	13

Fig. S4. Number of peptides identified on each SMX treatment at the end of the experiment belonging to each gene ontology (GO) molecular function category, according to UniPept Desktop1.2.1 tool analyses [12]. Protein abundance increases from red to green colour.

A) 50 µg L-1 (Day 17)

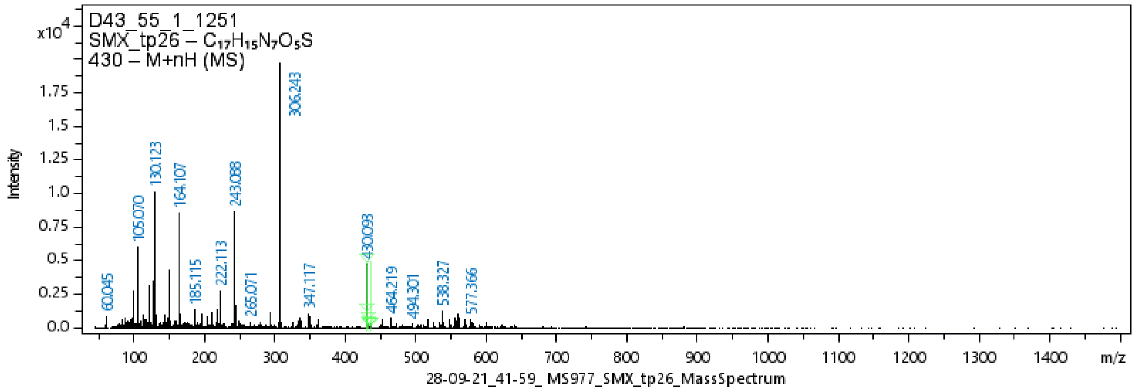

B) 50 µg L-1 (Day 25)

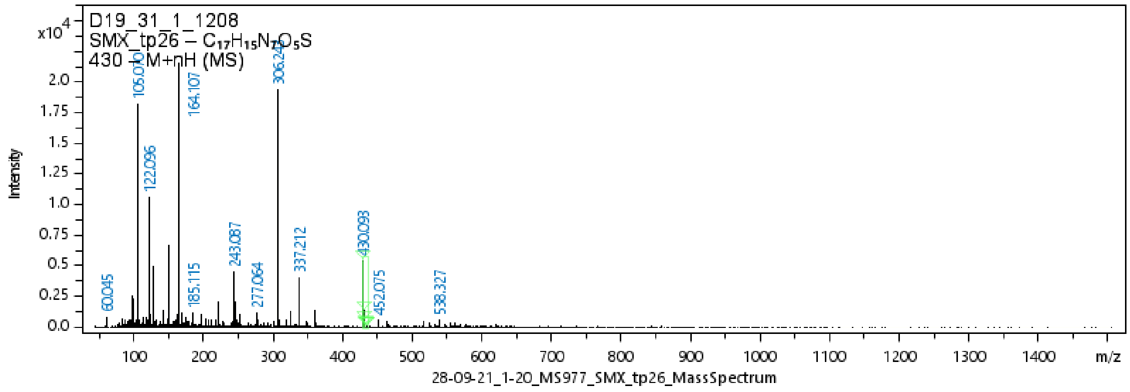

Fig. S5. Mass spectrums of the samples taken on days 17 and 25 from the bioreactors spiked with 50 µg L-1 SMX. The peaks in green correspond to PtO-SMX.

A) 250 µg L-1 (Day 2)

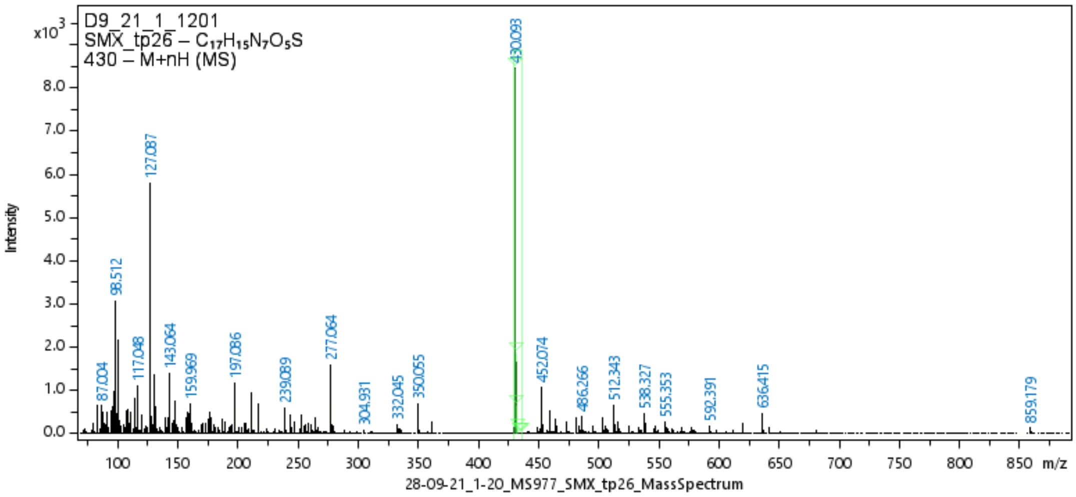

B) 250 µg L-1 (Day 17)

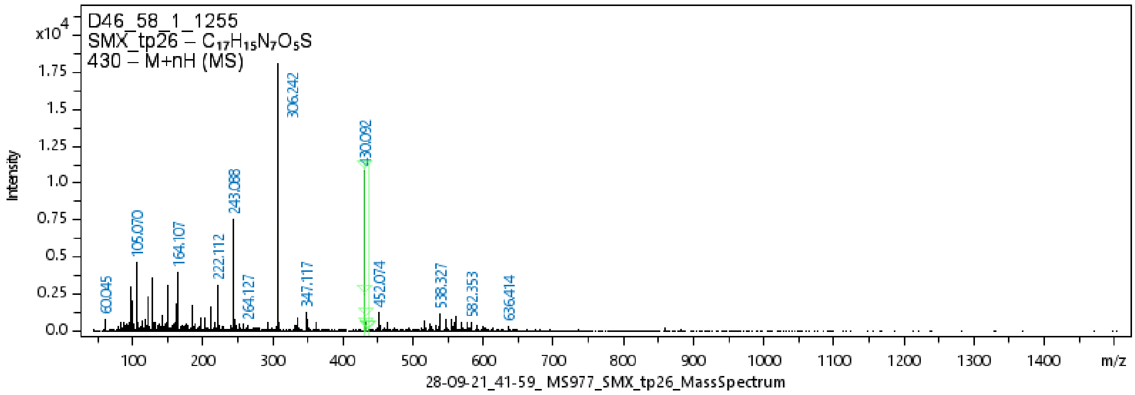

C) 250 µg L-1 (Day 25)

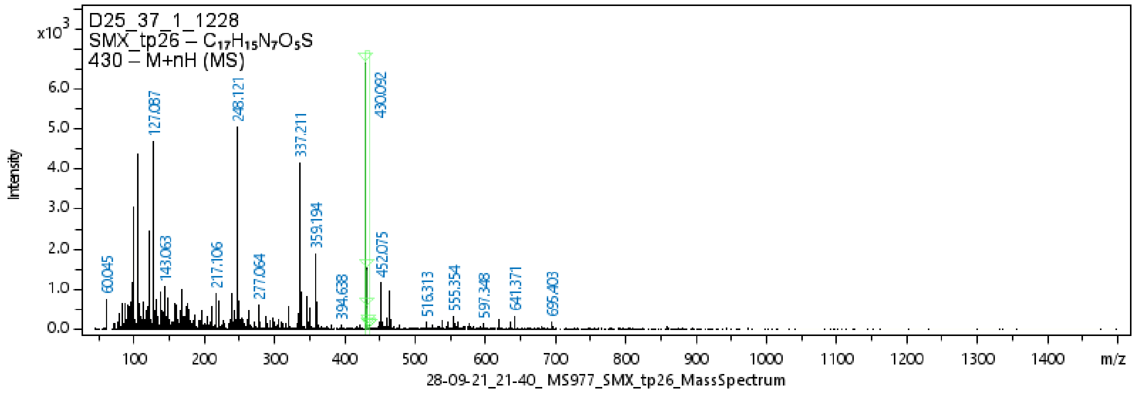

Fig. S6. Mass spectrums of the samples taken on days 2, 17 and 25 from the bioreactors spiked with 250 µg L-1 SMX. The peaks in green correspond to PtO-SMX.

A) 500 µg L-1 (Day 2)

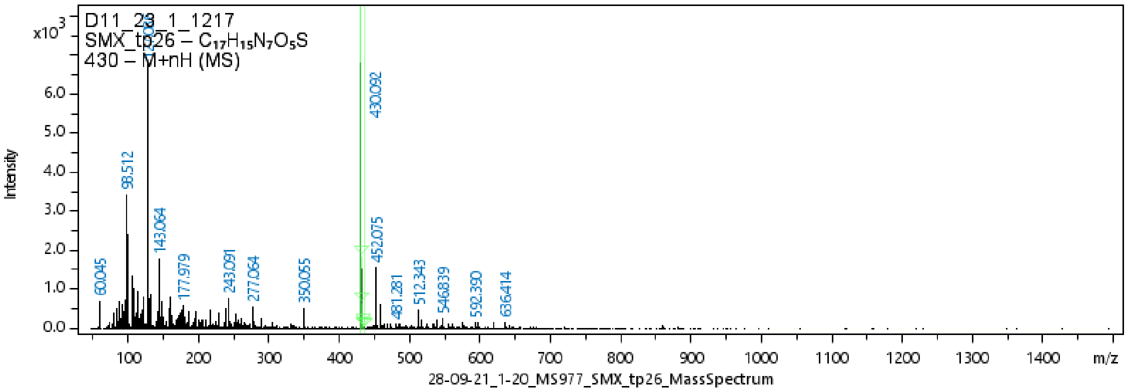

B) 500 µg L-1 (Day 17)

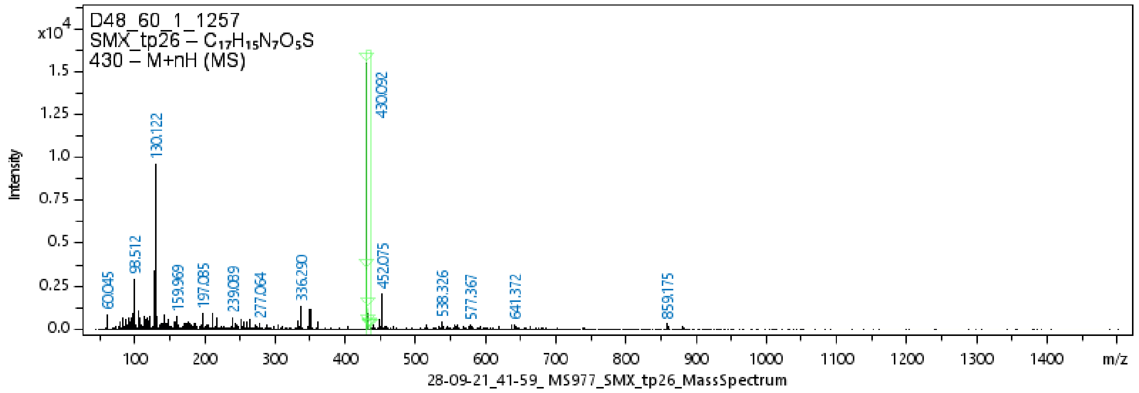

C) 500 µg L-1 (Day 25)

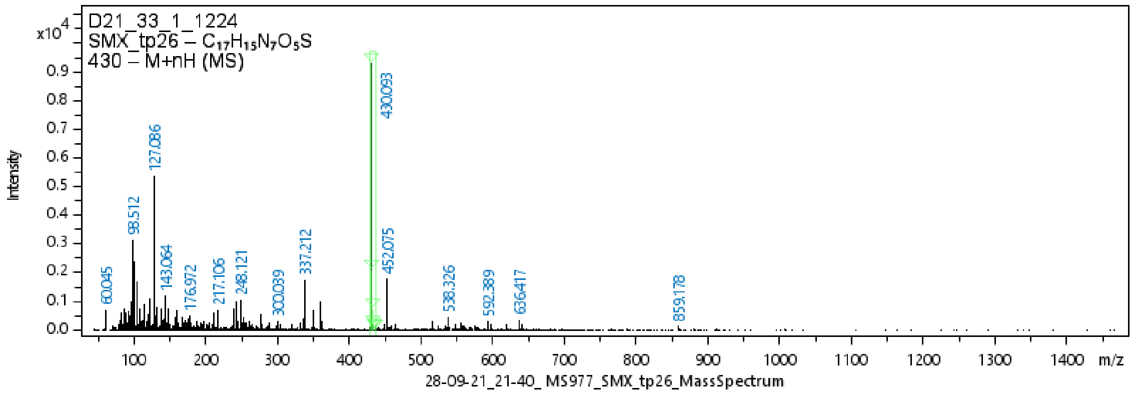

Fig. S7. Mass spectrums of the samples taken on days 2, 17 and 25 from the bioreactors spiked with 500 µg L-1 SMX. The peaks in green correspond to PtO-SMX.

A) 1000 µg L-1 (Day 2)

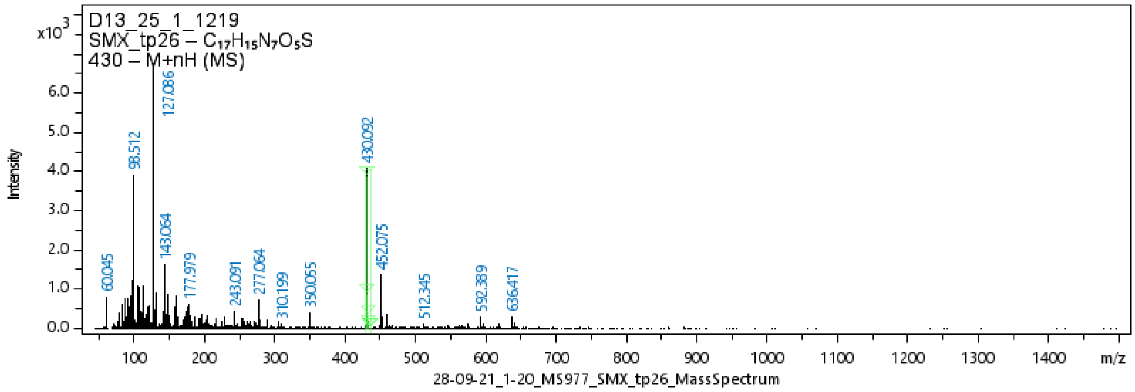

B) 1000 µg L-1 (Day 17)

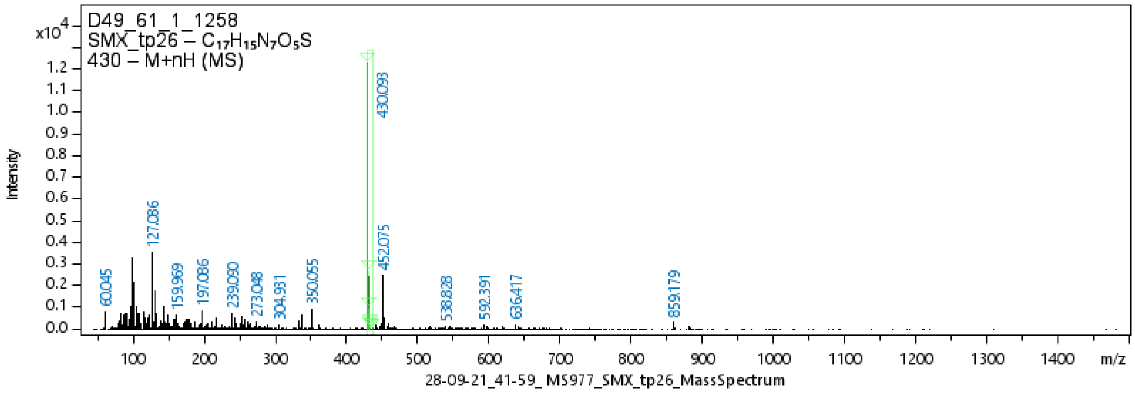

C) 1000 µg L-1 (Day 25)

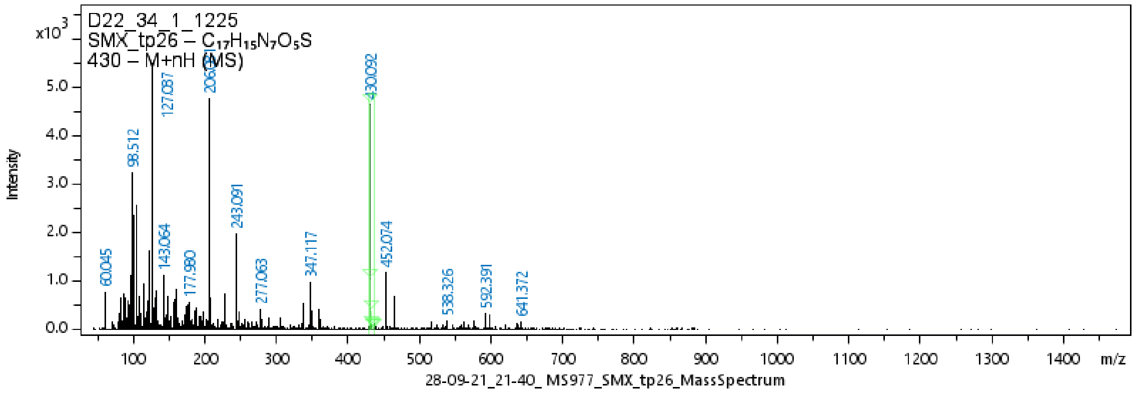

Fig. S8. Mass spectrums of the samples taken on days 2, 17 and 25 from the bioreactors spiked with 1000 µg L-1 SMX. The peaks in green correspond to PtO-SMX.

A) 2000 µg L-1 (Day 2)

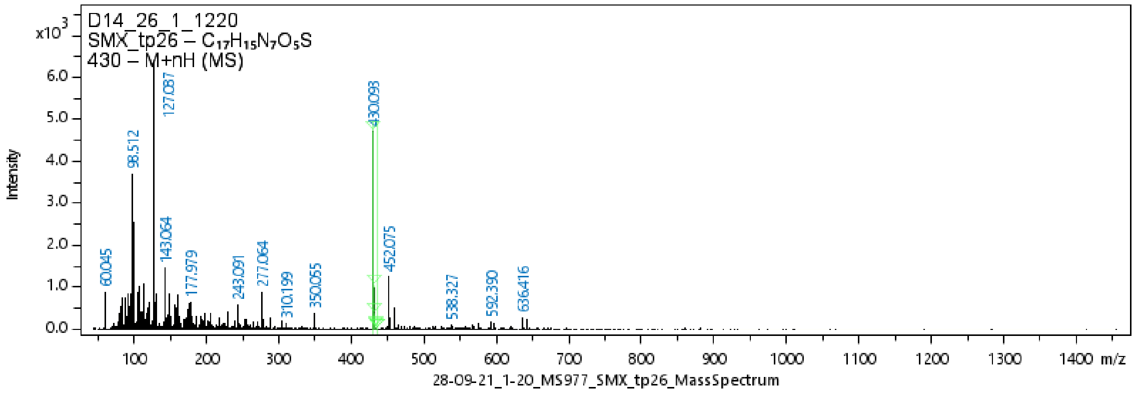

B) 2000 µg L-1 (Day 17)

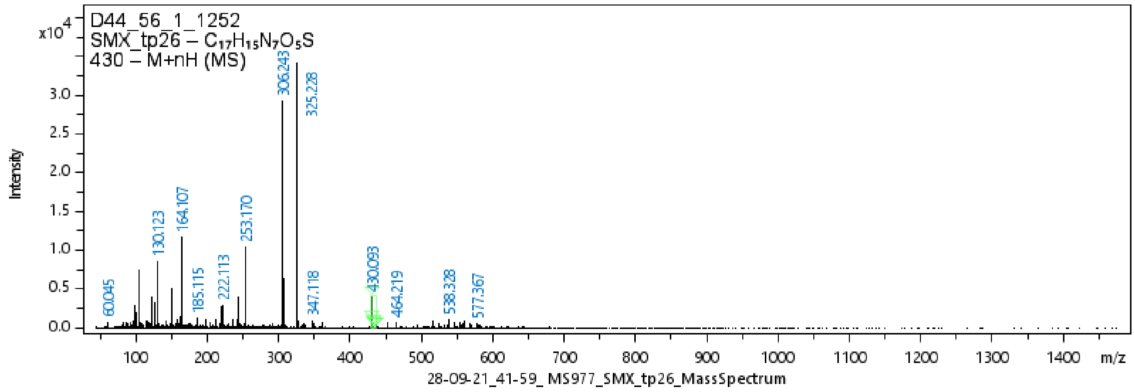

C) 2000 µg L-1 (Day 25)

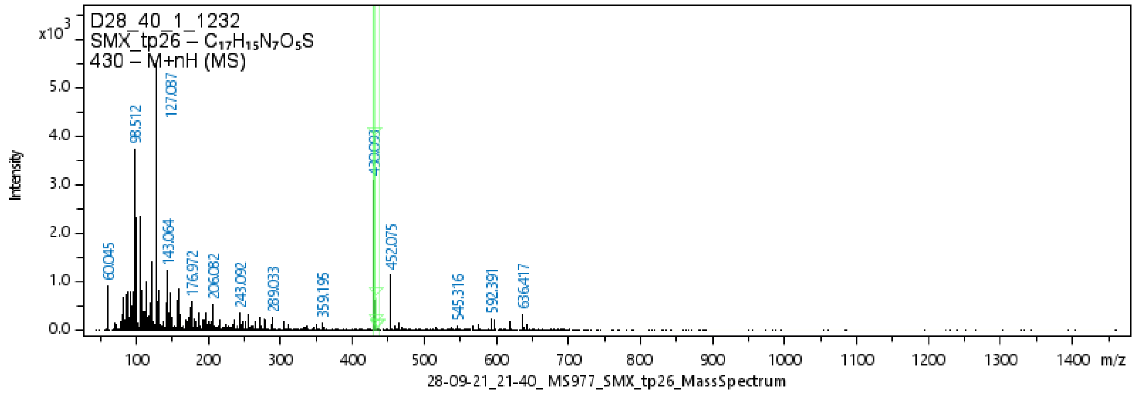

Fig. S9. Mass spectrums of the samples taken on days 2, 17 and 25 from the bioreactors spiked with 2000 µg L-1 SMX. The peaks in green correspond to PtO-SMX.

References:

(1) Wang, J., Wang, S., **2018**. Microbial degradation of sulfamethoxazole in the environment. Appl. Microbiol. Biotechnol. 102, 3573–3582.

(2) Mulla, S.I., Hu, A., Sun, Q., Li, J., Suanon, F., Ashfaq, M., Yu, C.P., **2018**. Biodegradation of sulfamethoxazole in bacteria from three different origins. J. Environ. Manage. 206, 93–102.

(3) Ricken, B., Kolvenbach, B.A., Bergesch, C., Benndorf, D., Kroll, K., Strnad, H., Vlček, Č., Adaixo, R., Hammes, F., Shahgaldian, P., Schäffer, A., Kohler, H.P.E., Corvini, P.F.X., **2017**. FMNH2-dependent monooxygenases initiate catabolism of sulfonamides in *Microbacterium* sp. strain BR1 subsisting on sulfonamide antibiotics. Sci. Rep. 7.

(4) Jiang, B., Li, A., Cui, D., Cai, R., Ma, F., Wang, Y., **2014**. Biodegradation and metabolic pathway of sulfamethoxazole by *Pseudomonas psychrophila* HA-4, a newly isolated cold-adapted sulfamethoxazole-degrading bacterium. Appl. Microbiol. Biotechnol. 98, 4671–4681.

(5) Nguyen, P.Y., Carvalho, G., Reis, A.C., Nunes, O.C., Reis, M.A.M., Oehmen, A., **2017**. Impact of biogenic substrates on sulfamethoxazole biodegradation kinetics by *Achromobacter denitrificans* strain PR1. Biodegradation 28, 205–217.

(6) Larcher, S., Yargeau, V., **2011**. Biodegradation of sulfamethoxazole by individual and mixed bacteria. Appl. Microbiol. Biotechnol. 91, 211–218.

(7) Achermann, S., Bianco, V., Mansfeldt, C.B., Vogler, B., Kolvenbach, B.A., Corvini, P.F.X., Fenner, K., **2018**. Biotransformation of Sulfonamide Antibiotics in Activated Sludge: The Formation of Pterin-Conjugates Leads to Sustained Risk. Environ. Sci. Technol. 52, 6265–6274.

(8) Kassotaki, E., Buttiglieri, G., Ferrando-Climent, L., Rodriguez-Roda, I., Pijuan, M., **2016**. Enhanced sulfamethoxazole degradation through ammonia oxidizing bacteria co-metabolism and fate of transformation products. Water Res. 94, 111–119.

(9) Gauthier, H., Yargeau, V., Cooper, D.G., **2010**. Biodegradation of pharmaceuticals by *Rhodococcus rhodochrous* and *Aspergillus niger* by co-metabolism. Sci. Total Environ. 408, 1701–1706.

(10) Reis, A.C., Čvančarová, M., Liu, Y., Lenz, M., Hettich, T., Kolvenbach, B.A., Corvini, P.F.X., Nunes, O.C., **2018**. Biodegradation of sulfamethoxazole by a bacterial consortium of *Achromobacter denitrificans* PR1 and *Leucobacter* sp. GP.

Appl. Microbiol. Biotechnol. 102, 10299–10314.

(11) Rabilloud, T. and Lelong, C., **2011**. Two-dimensional gel electrophoresis in proteomics: A tutorial. Journal of Proteomics, 74, 10, 1829-1841.

(12) Singh, R.G., Tanca, A., Palomba, A., Van der Jeugt, F.,Verschaffelt, P., Uzzau, S., Martens, L., Dawyndt, P., Mesuere, B, **2019**. Unipept 4.0: functional analysis of metaproteome data. Journal of Proteome Research, 18, 2, 606–615.
